# Isoquercetin as an Anti-Covid-19 Medication: A Potential to Realize

**DOI:** 10.3389/fphar.2022.830205

**Published:** 2022-03-02

**Authors:** Majambu Mbikay, Michel Chrétien

**Affiliations:** Functional Endoproteolysis Laboratory, Montreal Clinical Research Institute, Montreal, QC, Canada

**Keywords:** isoquercetin, quercetin, antiviral, coronavirus, SARS—CoV—2, COVID—19

## Abstract

Isoquercetin and quercetin are secondary metabolites found in a variety of plants, including edible ones. Isoquercetin is a monoglycosylated derivative of quercetin. When ingested, isoquercetin accumulates more than quercetin in the intestinal mucosa where it is converted to quercetin; the latter is absorbed into enterocytes, transported to the liver, released in circulation, and distributed to tissues, mostly as metabolic conjugates. Physiologically, isoquercetin and quercetin exhibit antioxidant, anti-inflammatory, immuno-modulatory, and anticoagulant activities. Generally isoquercetin is less active than quercetin *in vitro* and *ex vivo*, whereas it is equally or more active *in vivo*, suggesting that it is primarily a more absorbable precursor to quercetin, providing more favorable pharmacokinetics to the latter. Isoquercetin, like quercetin, has shown broad-spectrum antiviral activities, significantly reducing cell infection by influenza, Zika, Ebola, dengue viruses among others. This ability, together with their other physiological properties and their safety profile, has led to the proposition that administration of these flavonols could prevent infection by severe acute respiratory syndrome-coronavirus-2 (SARS-CoV-2), or arrest the progression to severity and lethality of resulting coronavirus disease of 2019 (Covid-19). *In silico* screening of small molecules for binding affinity to proteins involved SARS-CoV-2 life cycle has repeatedly situated quercetin and isoquercetin near to top of the list of likely effectors. If experiments in cells and animals confirm these predictions, this will provide additional justifications for the conduct of clinical trials to evaluate the prophylactic and therapeutic efficacy of these flavonols in Covid-19.

## Introduction

**FIGURE 1:**
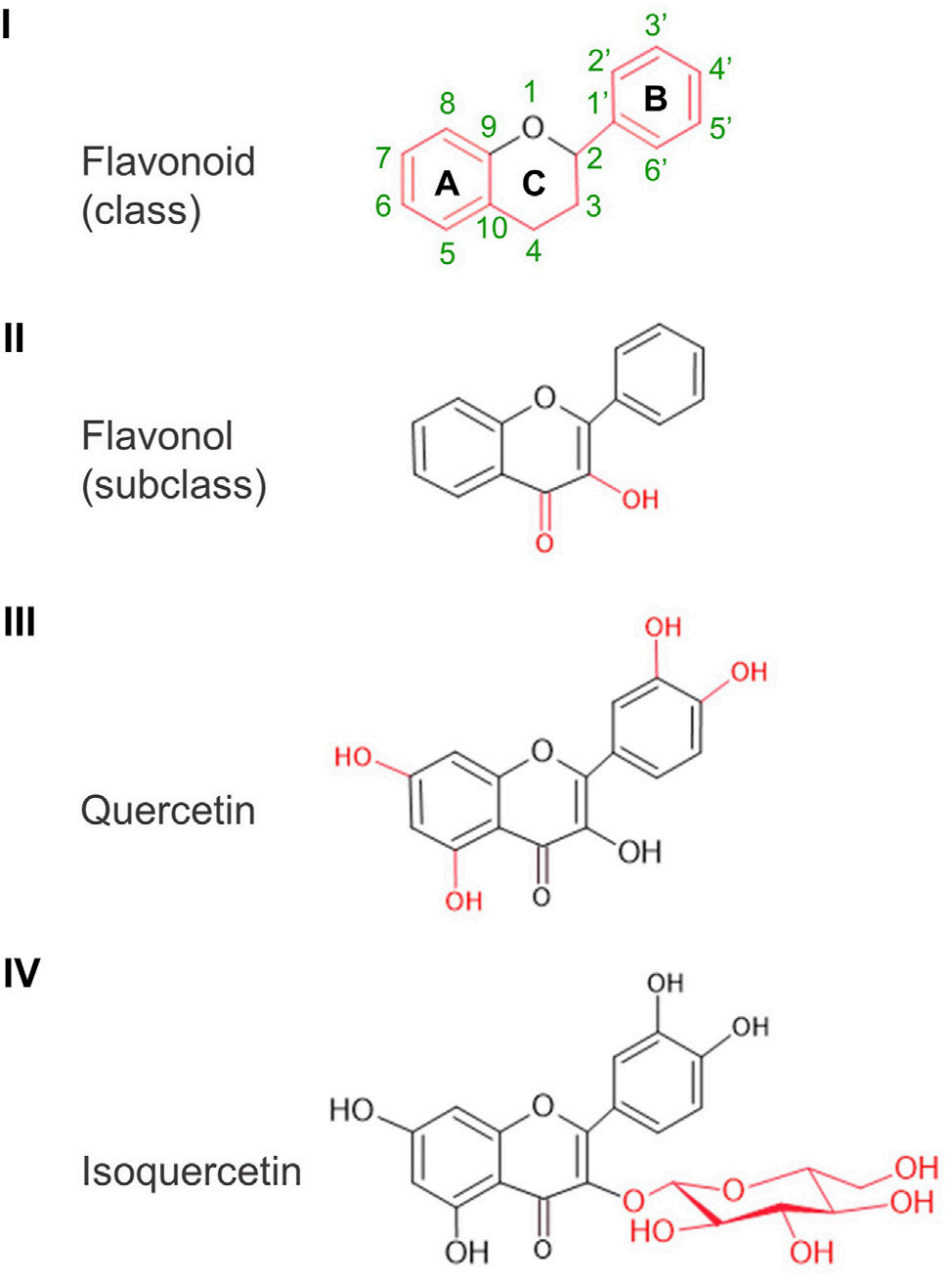
Basic structures: from flavonoid to isoquercetin. Class, subclass, and particular molecular features are illustrated in red. Atom numbering of the A, B, and C rings indicated for the basic structure of flavonoids applies to all the other structures.

Flavonoids form a widely diverse group of plant secondary metabolites which contribute to plant growth and survival in many ways, including germination and protection from environmental stresses (Kumar et al., 2018). As a common structural feature, they are made of 15 carbon atoms arranged in two phenolic rings (A and B rings) connected by a three-carbon chain in a C6-C3-C6 configuration. In flavonols, the three-carbon chain form a heterocyclic ketone ring (C ring) carrying a hydroxyl group on C3. Quercetin [IUPAC name: 2-(3,4-dihydroxyphenyl)-3,5,7-trihydroxychromen-4-one] is a flavonol; distinctively, it carries additional hydroxyl groups at C5 on the A ring as well as at C3′ and C4′ on the B ring (Figure 1).

Quercetin derivatives have one or more of these hydroxyl groups modified, often by sugars (e.g., glucose, galactose, rhamnose, arabinose, xylose), but also by methyl, sulfate, acetate, or phosphate groups. In its February 2020 report, the PubChem database counted 679 such derivatives^1^. They are ubiquitously and variably distributed in the plant kingdom, including in edible plant parts such as leafy vegetables, fruits, grapes, spices, and teas.

**FIGURE 2:**
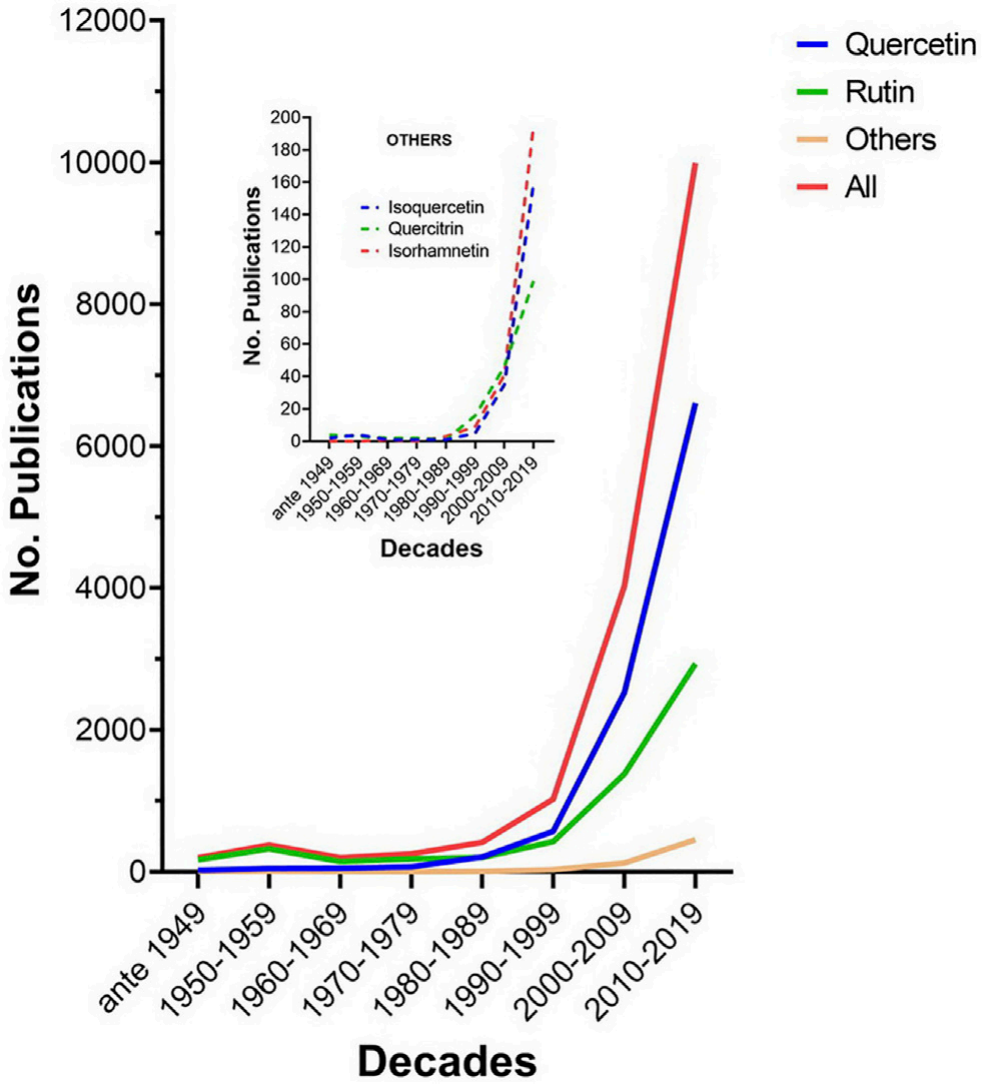
Publications per decade on quercetin and its major derivatives. Data were retrieved from Google Scholar using as separate keywords on the title page the terms quercetin aglycone, rutin, quercitrin, isorhamnetin, and isoquercetin (or isoquercitrin or quercetin-3-glucoside). The number of hits for quercetin aglycone, rutin, other derivatives, and the sum total were classified by decade. Other derivatives include quercitrin, isoquercetin, and isorhamnetin; their hits per decade are displayed in the inset.

Quercetin and its derivatives have been the object of intense scientific investigation in the last three decades as evidenced by the exponential growth in the number of scientific articles reported in Google Scholar. From less than 500/decade before the 1980s, these articles number close to 10,000 in the last decade (2010–2019) (Figure 2). The vast majority of studies were conducted with unmodified quercetin (quercetin aglycone). Derivatives which have also attracted the attention of investigators include quercetin-3-rhamoglucoside (rutin), quercetin-3-glucoside (isoquercetin or isoquercitrin), quercetin-3-rhamnoside (quercitrin), and 3’ methyl-quercetin (isorhamnetin).

In spite of the plethora of preclinical studies demonstrating the therapeutic potential of quercetins against various pathologies, including viral infections, efforts to convert this nutraceutical into a pharmaceutical for therapeutic use in humans has been frustrated by its poor bioavailability after ingestion. Many alternative forms of quercetin with a better metabolic outcome have been investigated, among them the natural mono-glycosylated isoquercetin and the manufactured poly-glycosylated enzymatically-modified isoquercetin (EMIQ) (Makino et al., 2009).

In this review, we focus on the distinctiveness of isoquercetin from quercetin aglycone. We first describe the general physiological properties of quercetin; then, relying mostly on studies that compare the two compounds in parallel, we examine how glycosylation influences these properties as well as the potential of isoquercetin as a better broad-spectrum antiviral for prophylactic and therapeutic use against SARS-CoV-2 infection and the resulting Covid-19 disease.

## Quercetin Restores Oxidative and Inflammatory Homeostasis

### Quercetin Against Oxidative Distress

Normal cellular metabolism involves reduction/oxidation (redox) reactions which result in the formation of free radical-generating atoms and molecules. Depending on whether the free radical is based on oxygen or nitrogen atoms, these metabolic byproducts are collectively known as reactive oxygen species [ROS, e.g., superoxide (O_2_^•−^), singlet oxygen (^1^O_2_), hydroxyl (^•^OH)] or reactive nitrogen species [RNS, e.g., nitric oxide (NO^•^), nitrogen oxide (NO_2_^•^)], respectively (Gupta et al., 2016). Examples of ROS/RNS-generating metabolic pathways include the electron transport chain of glucose oxidative phosphorylation which leads to the production of ATP in mitochondria (Zhao et al., 2019), the unfolded protein response (UPR) in the endoplasmic reticulum (ER) catalyzed by resident oxidoreductases, and the respiratory burst mediated in phagocytes in reaction to the presence of endogenous or exogenous ‘abnormal’ molecules (El-Benna et al., 2016).

All cells possess an endogenous antioxidant system that reduces these reactive species. Major components of the system involves glutathione (GSH) and associated reducing enzymes [e.g., glutathione peroxidase (GSH-Px), glutathione reductase (GSH-Rx) and superoxide dismutase (SOD)] and the protein cysteine thiol–disulfide exchange catalyzed by, among other enzymes, protein disulfide isomerase (PDI) (Gupta et al., 2016). When this system is overwhelmed, ROS/RNS react with biological macromolecules (e.g., lipids, proteins, carbohydrates, and nucleic acids), generating oxidized and dysfunctional varieties such as lipid peroxides, protein carbonyls, oxidized lipoproteins, In the words of Helmut Sies, the discoverer of hydrogen peroxide (H_2_O_2_), physiological ROS is associated with “oxidative eustress,” pathological ROS with “oxidative distress” (Sies, 2020).

Quercetin can efficiently counter oxidative distress. Its potent antioxidant activity of quercetin derives from the very low redox potential afforded by its multiple hydroxyl groups which allow it to donate electrons and protons to and capture electrons from ROS/RNS and other oxidized molecules. Quercetin also sustains the endogenous glutathione-based antioxidant system, stimulating reductases and inhibiting oxidases (Boadi et al., 2016). Moreover, by chelating transition metals (e.g., Fe^2+^, Cu^2+^), quercetin restricts their participation in oxidative processes and in ROS formation (Boots et al., 2008).

### Quercetin Against Inflammation

Inflammation involves a cascade of signaling events extending from plasma cell membranes to increased expression and nuclear translocation of transcriptional factors [e.g., nuclear factor kappa B (NF-κB), activating protein-1 (AP-1), nuclear factor-like 2 (Nrf-2)], to activation of a variety of genes for proinflammatory molecules, among them, the cytokines [e.g., tumor necrosis factor α (TNF-α)], interferons (INF), interleukins (IL), and chemokines. An immune response, when moderate and transient, is beneficial for tissue homeostasis. It is modulated by a balance between the two subsets of CD4^+^ T helper cells, Th1 cells which produce the proinflammatory TNFα and INF-γ, as well as IL-2 and 12 and Th2 cells which produce the anti-inflammatory IL-4, 5, 10, and 13. (Chatterjee, 2016). Quercetin is known to modulate the Th1/Th2 balance towards an overall cytokine profile that can induce a more effective and beneficial (i.e., non-pathogenic) immune response (Park et al., 2009; Tanaka et al., 2020).

### Quercetin Disrupts the Oxidation-Inflammation Feedback Loop

In the disease state, oxidative stress and inflammatory stress often feed on one another: on one hand, oxidized macromolecules are recognized by the innate immune system as damage-associated molecular patterns (DAMPs) (Chen et al., 2018); on the other hand, the stimulated immune system produces more free radicals to accelerate the destruction of cells exhibiting these patterns. This oxidation-inflammation feedback loop (OIFL) is the indiscriminate hallmark of a vast array of pathologies, including cancer, diabetes, atherosclerosis, hypertension, Alzheimer’s disease, and infections (Liguori et al., 2018; Furman et al., 2019). Quercetin appears to act as an “OIFL disruptor”. This ability might underlie many of its purported health benefits. Indeed, a number of preclinical studies have shown that quercetin can mitigate all the above-cited pathologies (Salehi et al., 2020).

## Isoquercetin is Quercetin Modified

Isoquercetin is generally considered a “pro-quercetin” in the sense that its biological activities follow its conversion to quercetin. This is largely true when isoquercetin is taken orally, but not when it is administered parenterally. Indeed, injected intravenously into rats, isoquercetin could be detected unaltered in 24-h urine (Choudhury et al., 1999). The question in this case is whether its physiologic activity is a true replication of that of its aglycone relative. Isoquercetin carries a single glucose moiety on carbon three of the C ring of quercetin backbone, losing an OH group (Figure 1). This modification not only renders it more hydrophilic and about 4-fold more soluble in water (∼206 μM) than quercetin (∼50 μM) (Makino et al., 2009), but also makes its B ring non-planar with the other two. These changes may influence its interaction of membrane lipid bilayers, its absorption and metabolism, as well as its physiological properties. As illustrated in the following paragraphs, *in vitro* and *ex vivo*, the glycosylated quercetin exhibits reduction of many of these properties compared to its aglycone relative; however, when taken orally, it provides a significant pharmacokinetic and physiological advantage, as it is better absorbed in the intestines and rapidly converted into the more active quercetin aglycone and its metabolites. The influence and impact of quercetin C3 glycosylation are described below.

### On Membrane Interactions

The first point of contact of quercetin or isoquercetin with the eukaryotic cell is the plasma membrane. The latter is made of a phospholipids bilayer to which are associated, incrusted or anchored, various lipids (e.g., cholesterol, triacylgycerols) and proteins (e.g., receptors, enzymes); and which is organized in dynamic functional microdomains (e.g., lipid rafts, caveolae, coated pits, ion channels). From and *via* the plasma membrane is initiated and propagated the intracellular transduction of signals that determine cellular physiology, including gene expression. It has been known since the 1970s that some pathologies are associated with significant alterations of membrane physicochemical properties (i.e., composition, fluidity, microviscosity, permeability). (Cooper, 1977; Scott, 1982). Some investigators attribute the pleotropic bioactivity of flavonoids, including quercetin derivatives, to their alterations of these properties (Tsuchiya, 2015). For example, by fluorescence polarization measurements on biomimetic membranes, quercetin affects lipid bilayer in biphasic fashion, fluidizing it at low concentrations (<2.4 μM) and rigidifying at higher concentrations (>5 μM) (Tsuchiya et al., 2002). At 10 μM, quercetin and isoquercetin differ in their impact on membrane fluidity: the former reduces it because of its greater hydrophobicity and deeper penetration within the lipid bilayer, whereas the latter, being more hydrophilic, does not (Tsuchiya, 2010). These differential interactions can variably affect the dynamics of membrane receptors, enzymes and other signaling molecules. Membrane fluidization is strongly associated with cancerous cell metastasis (Nicolson, 2015); inflammatory cell excitation (Calder, 2012), lipid peroxidation (Sergent et al., 2005), platelet aggregation (Vlasic et al., 1993), and infection by enveloped viruses (Harada, 2005). Thus, increased membrane rigidity afforded to cells by quercetins and other flavonoids may partly account for their broad spectrum antineoplastic, anti-inflammatory, antioxidant, antithrombotic, and antiviral properties.

### On Antioxidant Capacity and Activity

The antioxidant ability of a compound is assessed *in vitro* in term of capacity and activity: the former measures the end-point radical scavenging efficiency and potency; the latter the scavenging kinetics. These properties are strongly influenced by the steric structure of the compound as well as by the reaction solvent and its pH. It is generally measured on free radical-generating substrates such 1,1-diphenyl-2-trinitrophenylhydrazine (DPPH) and 2,2′-azino-bis(3-ethylbenzothiazoline-6-sulfonic acid ABTS) (Apak, 2019).

In cell-free assays, isoquercetin exhibits lower antioxidant capacity relative to quercetin in phosphate-buffered saline (PBS) at pH ≤ 6, and in methanol, but greater DPPH-measured activity in methanol (Xiao et al., 2021). This was also observed by Park et al. (2021) using the same flavonols. Interestingly, these investigators reported that, in HT22 mouse hippocampal cells, quercetin was about 10-fold more efficient than isoquercetin at reducing cellular ROS formation and the apoptosis that resulted from the treatment of these cells with 4 mM glutamate. Moreover, a combination of the two flavonols at concentrations that were individually ineffective against apoptosis, fully restored the viability of the glutamate-treated HT22 cells (Park et al., 2021), suggesting synergy between them.

Results obtained from cell-based antioxidant assays with these compounds may sometimes differ from those obtained from cell-free assays since, with cells, membrane characteristics and interactions come into play. For example, it have been established that membrane fluidity promotes lipid peroxidation on one hand, and that lipid peroxidation results in membrane rigidity, on the other hand (Borst et al., 2000). Thus, quercetin and isoquercetin may restore “healthy membrane fluidity,” by acting both as antioxidants and lipid bilayer-interacting molecules.

### On Anti-Inflammatory Property

*Ex-vivo*, the inflammatory inhibition of flavonoids is commonly evaluated using macrophages—primary or immortalized—stimulated with lipopolysaccharide (LPS) to produce more nitric oxide (NO) as a result of increased expression of inducible nitric oxide synthase (iNOS). Rat peritoneal macrophages pretreated with 100 μM quercetin and isoquercetin before LPS stimulation inhibited NO production by 66% and 48%, respectively, with a corresponding decrease in iNOS expression, indicating that the aglycone has a more potent anti-inflammatory activity (Lee et al., 2008). A differential reduction of these inflammatory marker was also observed in mouse macrophage RAW264.7 cells stimulated with LPS (Choi et al., 2012) or zymosan (Kim et al., 2013); the reduction was associated with inactivation of the NF-κB signaling pathway. In LPS-stimulated in BV2 mouse microglial cells, at 10 μM, quercetin was 6-fold more potent than isoquercetin at inhibiting NO production (Kwon et al., 2004). An alternative model cell system of inflammation-associated liver damage consists of human hepatocellular carcinoma HepG2 cells treated with 5% ethanol. Using this model, it was shown that, while a 1-h pretreatment with 10 μM quercetin or isoquercetin reduced to comparable extent ethanol-induced NO production and iNOS expression, the aglycone form were more effective than the glucoside at inhibiting TNFα secretion, as well as activation and nuclear translocation of the pro-inflammatory Nrf2 transcriptional factor (Lee et al., 2019).

*In vivo*, inhibition of inflammation by quercetin and isoquercetin seems comparable. For example, when air pouches generated subcutaneously on the backs of rats were injected with carrageenan, an inflammatory response ensued reflected by increased volume of the exudate as well of its content in cells, proteins, TNFα, prostaglandins and macrophage inflammatory protein 2. Injection of quercetin or isoquercetin (10 mg/kg) into the pouch 1 h prior to carrageenan challenge significantly and comparably reduced these inflammatory indices (Morikawa et al., 2003). Similarly, when mice immunologically primed by vaccination with ovalbumin were challenged intranasally with the same compound, they developed an asthma-like inflammatory reaction reflected by an increase, 24 h later, of leukocytes in bronchoalveolar lavage fluid, blood and pulmonary parenchyma. Oral administration of quercetin (10 mg/kg) and isoquercetin (15 mg/kg) similarly reduced eosinophil count in all three biological samples, suggesting they could be equally effective as anti-allergic drugs (Rogerio et al., 2007).

### On Anticoagulant Activity

Choi et al. (2016) conducted a battery of *in vitro*, *ex vivo*, and *in vivo* assays to comparatively evaluate the anti-coagulation activity of quercetin and isoquercetin. *In vitro* assays included fibrin clotting, fibrin polymer formation, thrombin activity, Factor Xa activity, and platelet aggregation, coagulation activated partial thromboplastin time (APTT) and prothrombin time (PT); the *ex vivo* assay consisted of measure of APTT and PT on blood collected after i.v. flavonol administration to mice; the *in vivo* assay consisted of evaluating the protection rate against thromboembolism induced by i.v. injection of human thrombin. By all these assays. The two flavonols were effective anticoagulants; *in vitro*, quercetin was more effective than isoquercetin, except in the Factor Xa inhibition assay; the effect of injected glucoside was stronger than of the aglycone in the *ex vivo* assay; it was slightly weaker in the *in vivo* assay. The greater effectiveness of the aglycone *in vitro* was also observed in an assay using platelet-enriched rat plasma treated with collagen to induce aggregation: 0.5, 1, and 2 mg/ml quercetin inhibited aggregation by 40, 100, and 100%, respectively; while, at the same concentrations, isoquercetin-induced inhibition was <10, 60, and 100%, respectively. (Ko et al., 2018).

Besides interacting directly with fibrin and thrombin, quercetin and isoquercetin can exert their anticoagulant effect through inhibition protein disulfide isomerase (PDI). This ER-resident oxidoreductase is also expressed in platelets and endothelial cells ending up at their surface; its expression, when upregulated by thrombin stimulation or vascular injury, could contribute to thrombogenesis (Xu et al., 2021). Strangely, when the flavonol inhibition of the reductase activity of recombinant PDI was assayed *in vitro* using a spectrometric measure of insulin aggregation in the presence of DTT, the IC_50_ of quercetin, isoquercetin, rutin, and quercetin-3-glucuronide, were >100, 7.1, 6.1, and 5.9 μM, respectively, indicating that these C3 modification in the quercetin derivatives enhanced the anti-PDI activity (Jasuja et al., 2012).

## The Pharmacokinetic Advantage of Isoquercetin

### From Improved Intestinal Absorption

In rats, orally administered isoquercetin is not absorbed and metabolized until it reaches the small intestine, whereas a fraction of quercetin can be taken up by the stomach and secreted into bile (Crespy et al., 2002). Quercetin aglycone is more lipophilic than isoquercetin: thus the aglycone, but not the glucoside, traverses the lipid bilayer and enters cells by passive diffusion as demonstrated by the rapid presence of it and its metabolites on the basolateral side of human intestinal Caco2 cells after application of the flavonols to the apical side (Murota et al., 2000). Penetration of the isoquercetin into cells is apparently facilitated by its hydrophilicity which leads to greater concentration near the intestinal brush border membrane where its sugar is removed by lactase phlorizin hydrolase, producing the aglycone which diffuses through the membrane (Day et al., 2000). The uptake also appears to be actively mediated by sodium-dependent glucose transporter 1 (SGLT-1). Indeed, in an *in vitro* mucosal uptake assay using pieces of rat jejunum, isoquercetin, but not quercetin, significantly inhibited SGLT-1-mediated uptake of a non-metabolisable glucose analogue in a competitive fashion, an inhibition potentiated by the addition of the SGLT-1 blocker phloridzin, indicating that the glucoside utilized the same transporter to enter cells (Ader et al., 2001; Wolffram et al., 2002). The sugar moiety of isoquercetin is removed by mucosal β-glycosylases as shortly as 30-min after perfusion of rat jejunum with isoquercetin, as only the aglycone form, its conjugates (mostly glucuronidated) and metabolites (mostly 3′ and 4′ methylated) could be found in the intestinal lumen and in blood veins (Morand et al., 2000a; Crespy et al., 2001; Chang et al., 2005).

### To Improved Pharmacokinetics and Pharmacodynamics

These mechanism of isoquercetin uptake by the intestinal mucosa may explain the 1.5-3-fold greater plasma concentration of quercetin and its metabolites after its ingestion compared to that of quercetin aglycone which has been observed in rats (Hollman et al., 1995; Morand et al., 2000a; Morand et al., 2000b), dogs (Reinboth et al., 2010), pigs (Cermak et al., 2003), and humans (Sesink et al., 2001). Comparing pharmacokinetic parameters after oral administration, isoquercetin yields a maximum plasma concentration C_max_) of quercetins 1.7 to 10-fold greater and the area under de curves for a given time (AUC_0-t_) 1.8 to 6-fold greater than quercetin aglycone, depending on species (Lesser et al., 2004; Makino et al., 2009; Reinboth et al., 2010; Stopa et al., 2017) (Table 1).

**TABLE 1:** Pharmacokinetic parameters of quercetin and isoquercetin after oral administration.

Species	Flavonol^a^	Dose	C_max_ ^b^	T_max_	AUC^a^ ^,^ ^b^_0–12*, 0–24**, 0-∞***_	References
Rat	—	μmol/kg bw	μmol/L	h	h × μmol/L*	Makino et al. (2009)
	Quercetin	50	0.26 ± 0.06	—	2.6 ± 0.7	—
	Isoquercetin	50	2.66 ± 0.81	—	15.8 ± 3.6	—
Dog	—	μmol/kg bw	nmol/L	H	min × μmol/L***	Reinboth et al. (2010)
	Quercetin	30	229.2 0.20	3.9 ± 0.5	174.9 ± 19.7	—
	Isoquercetin	30	888.3 ± 71	4.1 ± 0.3	410.2·± 26.7	—
Pig	—	μmol/kg bw	μmol/L	Min	min × μmol/L**	Lesser et al. (2004)
	Quercetin	30	0.518 ± 0.056	102.9 ± 8.0	117.3 ± 18.5	—
	Isoquercetin	30	0.908 ± 0.089	70 ± 7.9	205.5 ± 19.8	—
Man	—	mg/adult	μmol/L	H	h × μmol/L**	Stopa et al. (2017)
	Quercetin	500	0.8	—	3.8	—
	Isoquercetin	500	4.22	—	18.3	—

a Abbreviations: AUC, area under the curve; bw, body weight; IQC, isoquercetin; QC, quercetin aglycone.

b The quercetin forms titrated were: for rat and man, aglycone, glucuronidated, and methylated quercetins; for rat all (Makino et al., 2009) but methylated quercetins. Data are expressed a mean ±, standard error for rats and pigs, or standard deviation for dogs. -, not reported.

When tissue and plasma concentrations of quercetin metabolites (acid de-glucuronidated/de-sulfated quercetin as well as 3′ and 4′ O-methyl-quercetin) were measured after an 8-day oral gavage of ∼40 mmol/kg/d of quercetin and isoquercetin to rats, by comparison, isoquercetin gavage generated 2 to 5 more metabolites in tissues and 2 to 3 more in plasma than quercetin. The order of abundance in tissues was: lung > liver > kidney > heart cerebellum > cortex > hippocampus > striatum. The lung content of metabolites after isoquercetin gavage (10.1 nmol/g) was nearly 2.5 greater than that obtained after quercetin gavage (4.1 nmol/g) (Paulke et al., 2012).

The greater bioavailability of quercetin and its conjugates (which, as discussed below, are biologically active compounds on their own) after oral administration makes it more attractive for harnessing the health benefits attributed to quercetin aglycone.

## Isoquercetin is a Broad-Spectrum Antiviral

The potential of flavonoids, including quercetin, as broad-spectrum antiviral agents has been widely demonstrated by in cell lines and animal models, as recently reviewed (Badshah et al., 2021). Since isoquercetin, as a pro-quercetin, offers a better pharmacokinetics profile after oral administration, and therefore promises to be more efficacious, we examine here below experimental studies of its antiviral activity, in which the 50% inhibition concentration (IC_50_) and the 50% cytotoxicity concentration (CC_50_) were measured and have resulted in selectivity indices (SI = IC_50_/CC_50_) of ≥3 (Table 2).

**TABLE 2:** Isoquercetin antiviral efficiency and selectivity.

Virus	Strain/Isolate	Cell line	IC_50_ (μM)	CC_50_ (μM)	SI	References
IAV	Op	MDCK	1.2	46	38	Kim et al. (2010)
ZIKV	PRVABC59	Vero E6	1.2	>100	>83	Wong et al. (2017)
	PF-25013–18	Huh-7	14.0	>200	>14	Gaudry et al. (2018)
	PF-25013–18	A549	15.5	>200	>13	Gaudry et al. (2018)
	PF-25013–18	SH-SY5Y	9.7	>200	>21	Gaudry et al. (2018)
EBOV	Kikwit	Vero E6	5.3	>100	>19	Qiu et al. (2016)
	—	—	IC_50_ (μg/ml)	CC_50_ (μg/ml)	—	—
HSV	Types 1 and 2	Vero	0.4	>200	>500	Hung et al. (2015)
VZV	pOka	PFF	14.5	>20	>1.4	Kim et al. (2020)
HCMV	Towne	PFF	1.9	>20	>10	Kim et al. (2020)

Virus acronyms as well as species and organ origins of cells are described in the text. IC_50_, 50% inhibitory concentration; CC_50_, 50% cytotoxic concentration; SI, selectivity index.

### Against Influenza Virus

Influenza virus is an enveloped negative-strand RNA virus found in animals (e.g., birds, pigs) and humans and transmissible by air mostly through expiratory aerosol/droplet ejections during cough and sneezing. It causes typical symptoms of viral infections (i.e., fever, headache, muscle and joint pain) accompanied by respiratory discomforts (e.g., sore throat, cough, rhinitis) with possible complication of potentially lethal pneumonia. Although vaccines against it can provide relative protection, they have to be reformulated seasonally to counter new strains that result from the high mutation rate of its genome. Effective synthetic anti-IV drugs have been developed, but the eventual virus resistance to them justifies the search for novel drugs (Javanian et al., 2021).

Isoquercetin has the potential to become such a drug. In an assay using Madin-Darby canine kidney (MDCK) or green monkey kidney Vero cells, isoquercetin was shown to inhibit the replication of influenza A and B viruses (IAV and IBV) with an ED_50_ of 1.2 µM, 40-fold more effectively than quercetin aglycone (ED_50_ 48 µM). While serial passages of the virus in the presence of 2 µM of approved antiviral drugs, such as amantadine (a viral M2 ion channel inhibitor) or oseltamivir (a neuraminidase inhibitor), lead to the emergence of drug-resistant virus, passages in the presence of 2 µM of isoquercetin alone or with the above-cited drugs did not. When mice were intranasally infected with mouse-adapted IAV, the virus was detected in their lungs after 6 days, and the bronchial epithelium showed signs of necrosis. Daily i.p. injection of isoquercetin at 2 mg/kg/d and 10 mg/kg/d, starting 2 days pre-infection, resulted in 3.3-fold and 19.2-fold decrease in lung viral titer, respectively, and in less lung histopathological deterioration (Kim et al., 2010). The greater *in vivo* anti-IAV effectiveness of isoquercetin relative to quercetin was confirmed in a follow-up study (Thapa et al., 2012). Plaque formation by MDCK cells infected with avian H5N1 influenza virus in the presence of as low as 1 ng/ml quercetin and isoquercetin was also shown to be inhibited by 68 and 79%, respectively. (Ibrahim et al., 2013).

Mechanistically, isoquercetin did not inhibit neuraminidase, but blocked polymerase basic protein 2 (PB2) subunit of virus, while reducing oxidative and inflammatory stress as well PDI activity (Kim and Chang, 2018; Nile et al., 2019). Isoquercetin and mostly its quercetin-3-glucuronide were shown to bind PB2 with an affinity of −9.6 and −9.1 kcal/mol, respectively; and to inhibit its activity with a K_i_ of 3.7 and 0.2 μM, respectively (Gansukh et al., 2021).

### Against Zika Virus and Dengue Virus

ZIKV and DENV are single-strand positive RNA flaviviruses transmitted by mosquitoes. Besides systemic morbidity, infection by ZIKV can lead to neurological complications such as the Guillan-Barré Syndrome, encephalitis and microcephaly of the newborn; infection by DENV to fatal hemorrhagic fever and a shock syndrome (Silva et al., 2020). Because there is no drug to treat diseases caused by these viruses, the possibility that quercetin and its derivatives can counter the underlying infections came as a promising development.

We were the first to show that isoquercetin potently inhibited ZIKV infection and proliferation in Vero cells with an IC_50_ of 1.2 μM and IC_90_ of 1.5 μM, as measured by cytopathic effect and the level of the viral nonstructural protein NS1. Evaluating the *in vivo* anti-ZIKV efficacy of isoquercetin using immuno-compromised mice, we observed that, whereas all untreated mice succumbed to ZIKV infection after 7 days, 80% of mice i.p. injected with isoquercetin at 50 mg/kg/d survived after 7 days; and 50% of them after 30 days (Wong et al., 2017). The inhibitory effectiveness of isoquercetin against ZIKV infection depends in part on cell type. Thus, Gaudry and others (Gaudry et al., 2018) determined the IC_50_ in neuroblastoma SH-SY5Y, hepatocellular carcinoma Huh-7, and lung epithelial A549 cell line to be 9.7, 14.0, and 15.5 µM, respectively. In a series of elegant experiments using A549 cells, these investigators determined that isoquercetin did not affect viral particle integrity or virus attachment to cells, but inhibited virus internalization. Surprisingly, they found quercetin aglycone to be totally ineffective at inhibiting viral infection in these cells. In contrast, the aglycone was shown to be effective in Vero cells with an IC_50_ of 2.3 µM, and to inhibit the viral NS2B-NS3 protease *in vitro* with an ED_50_ of 1.17 µM (Zou et al., 2020).

*Ex vivo*, isoquercetin appears to be more effective against ZIKV than against DENV infection. DENV-2 infection of Vero cells have been shown to be inhibited by quercetin aglycone with IC_50_ around 30 μM (Zandi et al., 2011). *In vitro*, quercetin and isoquercetin reduced the activity of recombinant NS2B-NS3 protease of DENV-2 and DENV-3 with IC_50_ of 23 and 44 μM, and a K_i_ of 20 and 37 μM, respectively (De Sousa et al., 2015).

### Against Ebola Virus

EBOV is a filovirus which causes a high-fatality disease, called Ebola virus disease (EVD), transmissible through contact with infected biological materials and characterized initially by typical infective symptoms soon followed by severe gastrointestinal symptoms and, in some cases, coagulopathies and vital organ failure (Feldmann et al., 2020). The recent development of anti-EBOV therapeutic vaccines which efficaciously reduce EDV mortality does not preclude the need for alternative medications, in view of the possibility of emergence of immune-evading variants (Tshiani Mbaya et al., 2021).

Isoquercetin could be one such alternative. In 2016, we were the first to report that this flavonol could inhibit infection of Vero cells by distinct species of EBOV with an IC_50_ and IC_90_ of 5.3 μM and 9.3 μM, respectively. Intraperitoneal (i.p.) injection of isoquercetin at 50 mg/kg into mice as little as 30 min prior to infection with lethal dose of mouse-adapted EBOV, followed by similar i.p. injections of the drug every other day, protected 90%–100% animals from EDV mortality. This protection was associated with dramatic reduction of viral load in tissues and blood. Initiation of treatment 1 day post-infection led to only 30% protection, indicating that the flavonol is most effective as a prophylactic anti-EBOV drug (Qiu et al., 2016). The proposed mechanisms of the anti-EBOV activity of isoquercetin involves inhibition of viral entry (Qiu et al., 2016) as well as maintenance of the cellular antiviral interferon signaling cascade which can get blocked by the EBOV protein VP24 in the early steps of infection (Fanunza et al., 2020).

### Against Coronaviruses

Coronaviruses are enveloped positive RNA-strand viruses of the *Coronaviridae* family which infects animals and men and are transmissible across species. Their genome encodes several proteins, the most druggable being the RNA-dependent RNA polymerase (RdRp), the chymotrypsin-like protease 3 (3CL^pro^), the papain-like protease (PL^pro^), the envelope spike (S) glycoprotein. Coronavirus that infect humans can cause diseases ranging from mild upper respiratory disease to severe respiratory syndromes. Recent outbreaks of severe syndromes were caused by severe acute respiratory syndrome-coronavirus-1 (SARS-CoV) in 2003, Middle East respiratory syndrome-coronavirus (MERS-CoV) in 2012, and SARS-CoV-2, the cause of the ongoing pandemic of coronavirus disease since 2019 (Covid-19). Although effective anti-SARS-CoV-2 vaccines have been developed and are being deployed around the world, the need for broad-spectrum therapeutic drugs against immune-evading variants of the virus remains nonetheless.

Since the above outbreaks, there has been a flood *in silico* molecular docking analyses and molecular dynamics simulations of flavonoids binding to target viral proteins. In one such study, quercetin and isoquercetin showed strong affinity (dock score −7.7 to −10.3 kcal/mol) for SARS-CoV-2 S protein, RdRp, 3CL^pro^ and PL^pro^, suggesting that these compounds may possess anti-CoV efficacy (Hiremath et al., 2021). Experimental evidence seems to support some of the *in silico*-derived inferences. For example, *in vitro* assays using recombinant 3CL^pro^ from SARS-CoV or MERS-CoV and a Förster resonance energy transfer (FRET) substrate have shown that quercetin or isoquercetin can inhibit the hydrolytic activity of these proteases with IC_50_ in the 24–73 μM range (Ryu et al., 2010; Nguyen et al., 2012; Park et al., 2017; Jo et al., 2019); and that quercetin can inhibit recombinant SARS-CoV PL^pro^, with an IC_50_ of 8.6 μM (Park et al., 2017). FRET assays were also used to screen a 150 compound-library of small molecules for inhibition of recombinant SARS-CoV-2 3CL^pro^: with an inhibition constant (Ki) of ∼7 μM, quercetin was found to be the most potent inhibitor. Furthermore, by isothermal titration calorimetry, it was determined that the flavonol binds to the protease with a dissociation constant (K_d_) of 2.7 and 10 μM, in the presence of 0 and 150 mM NaCl, respectively (Abian et al., 2020). Quercetin was found to inhibit SARS-CoV-2 replication in Vero cells with an IC_50_ of 192 μM (Mangiavacchi et al., 2021).

### Against Other Viruses

Isoquercetin has also been shown to inhibit herpesviruses *ex vivo*: herpes simplex virus type 1 (HSV-1) and HSV-2 (Abou-Karam and Shier, 1992; Chen et al., 2011) as well as varicella-zoster virus (VZV) and human cytomegalovirus (HCMV) with IC_50_ of 15 and 2 μM, respectively (Kim et al., 2020).

## Isoquercetin Against Covid-19

### Pathophysiology of Covid-19 in Brief

Covid-19 is initiated through infection of airway epithelial cells by SARS-CoV-2. The entry of the virus follows attachment of the S glycoprotein of the viral envelope to angiotensin converting enzyme 2 (ACE2) at the surface of the cells. The attachment and fusion is facilitated by host cell surface proteases such as the proprotein convertase furin, transmembrane protease serine 2 precursor (TMPRSS2) as well as endo/lysosomal cysteine protease cathepsins L (CatL). After endocytosis, the virus is uncoated in endosomes and its RNA released in the cytoplasm where it is used by the host translational machinery to proteins necessary for the replication and multiplication of the virus (e.g., RdRp, 3CL^pro^, and PL^pro^), causing damage to the airway epithelium and resulting in respiratory distress. The progeny viruses are released in the bloodstream, whence they infect all organs expressing the ACE2 receptor, including the liver, the intestine, the kidney, the heart, and the brain (Harrison et al., 2020; Zhao et al., 2021).

Physiologically, tissue damages induce a robust inflammatory response involving recruitment of helper cells, macrophages and monocytes, increased secretion of proinflammatory cytokines (e.g., INF-γ, TNFα, IL1β, IL-2, IL-6, IL-12) and chemokines (e.g., MCP-1), increased membrane permeability, and increased expression of adhesion molecules. The inflammatory response is very probably accompanied by reciprocally-sustaining oxidative stress. This oxidation-inflammation feedback loop could be one of the causes of the immune hyper-responsiveness dubbed “cytokine storm”. The “storm,” acting on platelets and vascular endothelium induces coagulopathies (e.g., venous thromboembolism, disseminated intravascular coagulation) which contribute to fatal multi-organ injury and failure (Harrison et al., 2020; Savla et al., 2021).

### Isoquercetin Targets During Covid-19 Pathogenesis

**FIGURE 3:**
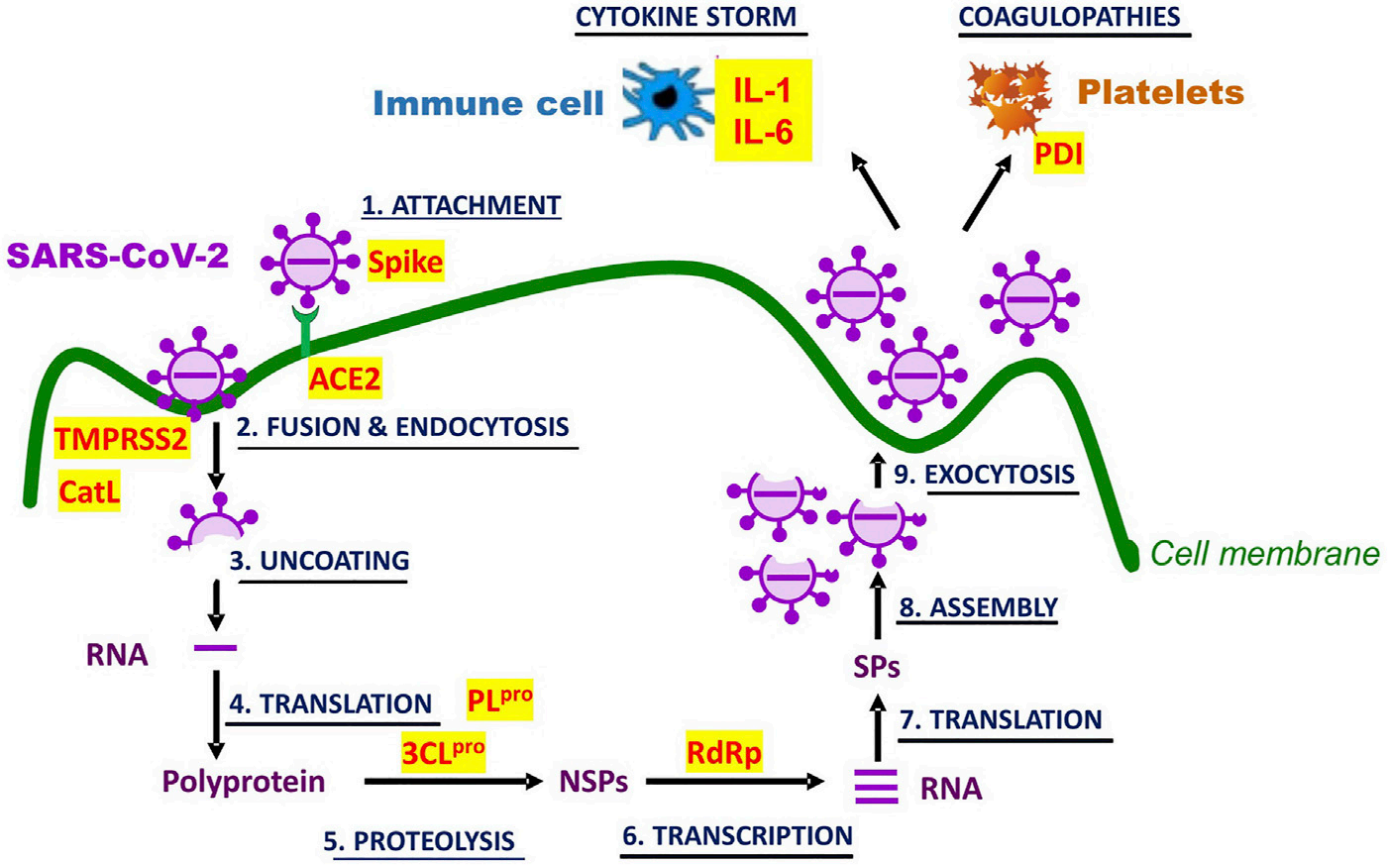
Molecular and physiological targets of quercetin and isoquercetin in Covid-19. SARS-CoV-2 infection and life cycle are graphically represented. Potential targets for inhibition by the flavonols are written in red and highlighted in yellow. Their full identity and the role in Covid-19 pathogenesis are described in the text. NSPs stand for non-structural proteins, and SPs for structural proteins.

Isoquercetin and/or quercetin aglycone, could act on several targets to oppose SARS-CoV-2 infection and halt the pathological course of Covid-19. Possible targets are molecular and physiological (Figure 3). Among molecular targets, there is not only the viral 3CL^pro^ and PL^pro^ whose inhibition by quercetins has been experimentally demonstrated (Section 5.2), but also the host ACE2, TMPRSS2, and CatL. To investigate interaction of flavonols with the ACE2 receptor Zhan et al. (2021) fixed the membrane fraction of HEK293 cells overexpressing human ACE2 (HEK293-ACE2^h^) to carboxymethylcellulose (CMC); of this resin, they make columns which they used in liquid chromatography to show that quercetin and its methylated metabolite isorhamnetin specifically bound to ACE2^h^-CMC. In a surface plasmon resonance assay, they also determined that quercetin and isorhamnetin bound to ACE2 with K_d_ of 5.9 and 2.5 μM, respectively. They further tested whether the binding could be of consequence for SARS-CoV-2 Spike pseudotyped virus infection of HEK293-ACE2^h^ cells and observed that, at 50 μM, only isorhamnetin inhibited infection by 48%. As for the other host proteases, CatL was shown to be inhibited by quercetin and isoquercetin with an IC_50_ of 26.3 μM and 115 μM, respectively (Ramalho et al., 2015); on the other hand, by *in silico* analysis, TMPRSS2 can bind quercetin with an affinity of −−7.7 kcal/mol (Alzaabi et al., 2021), suggesting that its protease activity might be diminished by this flavonol. Although quercetin interaction with each one of these molecular targets is moderate (in the micromolar range), the multiplicity of targets may accentuate the antiviral effectiveness of the flavonol.

Physiological targets may include pathogenic manifestations of viral infections which quercetins are known to counter, most damaging among them being oxidative stress, hyperactive inflammation, and coagulopathies. As of now, the improvement conferred by quercetins in these manifestations are inferred mostly from preclinical studies of infections by virus other than SARS-CoV-2. Quercetin protection against inflammation-induced coagulopathy and multi-organ injury was examined in a rabbit model generated by continuous infusion of 100 μg/kg/h LPS for 6 h: the treatment caused inflammation (↑TNF-α), increased coagulability (↑APTT, ↑PT, ↓fibrinogen, ↓protein C, ↓antithrombin III), as well as kidney (↑blood urea nitrogen), and liver (↑alanine aminotransferase) dysfunctions. Intravenous administration of quercetin (0.5, 1.0, and 2.0 mg/kg/h) dose-dependently and significantly attenuated these alterations of hemostatic and functional parameters (Yu et al., 2013).

### Concerns on the Use of Quercetin and Isoquercetin as Anti-Covid-19 Drugs

From experimental studies with cells and animals, there is ample evidence that quercetin aglycone possesses physiological activities that could promote or restore health. Its safety at therapeutic doses has been established in animals and humans (Harwood et al., 2007), leading to its qualification by the US Drug and Food Administration (FDA) as a Generally Recognized as Safe (GRAS) compound (US FDA, 2010). Isoquercetin shows an equally favorable safety profile (Hobbs et al., 2018). In phase 1 clinical trials, no drug-linked severe adverse effects were reported with quercetin up to 5 g/d (Lu et al., 2016) and isoquercetin up to 1 g/d (Buonerba et al., 2018; Zwicker et al., 2019).

Some of the most cited arguments against its utility as a pharmaceutical in humans have been its poor absorption and bioavailability due in part to its sequestration by serum albumin (Fiorani et al., 2003), its rapid metabolization, and its interindividual variability as illustrated by the wide C_max_ coefficient of variation (CV: 37–96%) of quercetin and its metabolites in the plasma of healthy volunteers who had ingested ∼1.1 g quercetin aglycone (Guo et al., 2014).

Oral isoquercetin exhibits a significantly improved bioavailability by nearly 5-fold over quercetin aglycone (Stopa et al., 2017) (Table 1). Moreover, isoquercetin has a 17-fold weaker affinity for albumin than the aglycone form (Martini et al., 2008), suggesting that it is less likely to be sequestered by albumin or other binding proteins in the intestinal mucosa and in blood. Interestingly, Fiorani et al. (2002), Fiorani et al. (2003) have shown in *ex vivo* assays that quercetin accumulates in red blood cells (up to 0.4 mM), imbedded into membranes or bound to hemoglobin, and that albumin can extract it from these cells, an indication that, *in vivo*, the serum protein could serve as a circulating carrier of the flavonol and its distributor to tissues.

Concerning the rapid metabolization of isoquercetin once deglycosylated, it should be noted that some of its major metabolites such as quercetin-3-glucuronide and isorhamnetin exhibit many of the physiological properties of the aglycone, including antiviral properties (Fan et al., 2011; Terao et al., 2011; Gong et al., 2020). Furthermore, glucuronidated quercetin could also serves as a precursor to the aglycone form, as it is susceptible to deconjugation by cellular β-glucuronidase whose level is up regulated by inflammatory states (Terao et al., 2011). Thus, the effective concentration of quercetin *in vivo* could be far greater than that estimated on the basis its plasma levels only.

Interindividual variability in plasma quercetin after ingestion has been partly attributed to differences of intestinal permeability. Increased permeability has been associated with higher levels of circulating bacterial endotoxin. Plasma quercetin level has been found to positively correlate with that of plasma endotoxin (Guo et al., 2014). Intestinal permeability is strongly influenced by the intestinal microbiome (Chakaroun et al., 2020). Isoquercetin and quercetin have been reported to reshape the microbiota of mice fed a high-fat diet while reducing the metabolic syndrome induced by the diet (Tan et al., 2018; Tan et al., 2021). One can speculate that the beneficial effect of quercetin on the syndrome results in part from the reduction of intestinal permeability due to microbiome reshaping. This reduction probably lowers quercetin absorption while maintaining pharmacokinetics variability due to alternate causes (e.g., genetics, age, lifestyle, health conditions) (Lin et al., 2021). Ultimately, one must wonder whether such variability matters for biological function, especially if the effective concentration in tissues is on the lower side of the concentration range. It worth noting that when quercetin or isoquercetin was fed to rats, mice or pigs, its metabolites accumulated primarily in tissues that are known to be susceptible to SARS-CoV-2 infection, namely lung, liver, intestine, kidney and heart, although the order of abundance per tissue differs among species (De Boer et al., 2005; Paulke et al., 2012; Liu et al., 2014). This accumulation of metabolites in relevant tissues suggests that effective levels against infection by the virus may readily be reached in these sites.

## Conclusion and Perspective

In this review, we presume that, as a potential anti-SARS-CoV-2 drug, isoquercetin represents a better choice than quercetin aglycone because it is a more absorbable precursor of quercetin, allowing greater bioavailability of the aglycone. Together, these two flavonols represent potentially effective medications for the treatment of COVID-19 patients, the reasons being: 1) their broad-spectrum antiviral activities; 2) their potent activities against SARS-CoV-2-induced symptoms as an antioxidant, anti-inflammatory, immunomodulatory, anticoagulant agent; 3) *in silico* analyses by molecular docking and molecular dynamics simulations, indicating that several proteins involved in SARS-CoV-2 entry and replication exhibit strong affinity for these flavonols; 4) their safety when orally administered in phase 1 clinical trials. Experiments *in vitro* and in animals should corroborate this presumption and elucidate their mechanisms of their action.

At the writing of this review, nine phase-2 clinical trials evaluating the efficacy of quercetins against Covid-19 have been registered in the National Institute of Health database.^2^. Four trials are listed as completed; the results of one trial have been published in a peer-reviewed journal (Di Pierro et al., 2021). In this trial, a formulation called quercetin phytosome® containing, as additive, the absorption-enhancing sunflower lecithin (Riva et al., 2019), was given orally (1.7 g/day for 7 days and 1 g/day for the subsequent 14 days) along with recommended standard care to middle-aged patients (*n* = 21) in the early stage of Covid-19; an equal number of patients of similar average age and disease stage was given standard care only. Besides clinical symptoms, RT-PCR-detectable positivity for SARS-CoV-2 RNA in nasopharyngeal swabs was monitored weekly. This positivity declined far more rapidly in the quercetin-treated group (*p* = 0.0002) and this was associated with greater symptomatic improvement (*p* = 0.012), suggesting that quercetin may promote viral clearance in SARS-CoV-2-infected subjects and accelerate their recovery. The positive results of this formulation need to be corroborated by larger studies with more extended array of clinical and laboratory parameters. They call for the evaluation of isoquercetin formulations also as potential anti-Covid-19 medication.

## Footnotes

1
https://pubchem.ncbi.nlm.nih.gov/

2
https://clinicaltrials.gov
